# Environmental DNA size sorting and degradation experiment indicates the state of *Daphnia magna* mitochondrial and nuclear eDNA is subcellular

**DOI:** 10.1038/s41598-019-48984-7

**Published:** 2019-08-29

**Authors:** Rashnat Moushomi, Gregory Wilgar, Gary Carvalho, Simon Creer, Mathew Seymour

**Affiliations:** Molecular Ecology and Fisheries Genetics Laboratory, School of Natural Sciences, Bangor University, Bangor, Gwynedd LL57 2UW UK

**Keywords:** Freshwater ecology, Molecular ecology

## Abstract

Environmental DNA analysis has emerged as a key component of biodiversity and environmental monitoring. However, the state and fate of eDNA in natural environments is still poorly understood for many ecological systems. Here we assess the state and fate of eDNA derived from the water flea, *Daphnia magna*, using a full factorial mesocosm experiment. We measured the quantity and degradation of eDNA over a two month period across a range of filters differing in pore size (0, 0.2, 1 and 10 µm), which spans the range of eDNA source material including subcellular, cellular and tissue. We also used two primer sets targeting mitochondrial (COI) and nuclear (18S) genomic regions. Our findings demonstrated that eDNA was most prevalent in the effluent water, but also reliably detected on the 0.2 μm filter, suggesting subcellular material is the predominate state of eDNA. Temporal eDNA quantity dynamics followed an exponential decay function over the course of 6-17 days, demonstrating a predictable decline in eDNA concentration. Nuclear eDNA was more abundant than mitochondrial eDNA, which may be a result of greater primer affinity, or indicate greater availability of nuclear eDNA gene targets in the environment. In contrast to two previous size-sorting experiments, which utilizing fish eDNA, our findings suggest that the state of invertebrate eDNA is much smaller than previously suspected. Overall, our data suggest that the detection of eDNA greatly depends on our knowledge of the state and fate of eDNA, which differ among species, and likely across environmental conditions.

## Introduction

There is no other source of biodiversity information like environmental DNA (eDNA), which can detect whole communities from any environment. Consequently, eDNA analysis has been named as a ‘game changer’ for biodiversity sampling^1,2^. Environmental DNA refers to macrobial DNA that is extracted from an environmental sample (e.g. water, soil or air), without targeting a particular organism^3^. Combined with genetic amplification and sequencing, eDNA analysis enables a wide range of research questions, across a wide range of disciplines; including molecular ecology, palaeontology, conservation/invasion biology, ecology and environmental sciences^4,5^. The non-invasive means of detecting species via eDNA provides a reliable biomonitoring approach that avoids disturbing the ecology of the target species^3,6,7^. In case of aqueous environments, eDNA of macro-organisms offers a simple and sensitive standardized means of sample collection and identification, often with better detection of diverse fauna, compared to traditional direct sampling methods^8,9^. Aquatic eDNA has been utilized for monitoring ecological communities^10,11^, assessing invasion dynamics^12^, conservation monitoring^13^ and assessing localized extinction^14^. Furthermore, eDNA can be collected from any type of aquatic environment, and has been used to assess species living in lakes, rivers, groundwater and marine environments^11,15–17^. Though eDNA stands as an important tool to complement traditional methods, there are still some shortcomings in our understanding of the very nature of eDNA that can impede practical eDNA study designs^18,19^.

The ability to relate eDNA information to its original source, either a target species or whole community diversity, is limited by our understanding of the temporal, physical, and chemical factors that influence eDNA detectability in natural environments. The detection of diverse aquatic macrofauna using eDNA requires the knowledge of origin (e.g. physiological source), state (e.g. intra- or subcellular particles), fate (e.g. suspension time) and transport dynamics of eDNA^20^. The state of eDNA is an important domain that directly affects the ability of current sampling methods to detect eDNA. Our current understanding is that eDNA state predominates as necromass (dead biomass), of various concentrations, in water, soil and sediments^21,22^. In aqueous environments, eDNA necromass can remain in many forms such as intracellular DNA, subcellular DNA, soluble DNA, non-soluble DNA, DNA within dead but structurally intact cells, organically or inorganically complex DNA and DNA absorbed by sediment minerals^23^. Structurally, eDNA is found in the environment in both intracellular (e.g. tissue or cells) and subcellular (e.g. mitochondria, ribosomes or free floating nucleotide strands) forms with intracellular eDNA transforming to subcellular eDNA over time as cells degrade^24^.

What remains unclear is what eDNA state (intracellular or subcellular) dominates an environment when eDNA sampling takes place. Present aqueous eDNA sampling methods primarily utilize a range of water filtering approaches, with limited consideration for the particle size (i.e. state) of the targeted eDNA of interest. The range for nominal filtering for eDNA studies varies widely across taxonomic groups, ranging from <0.2 μm to ≥180 μm^25–30^. However, eDNA particle sizes likely differ among species and bodily sources of origin, such as eDNA originating from whole body decomposition versus eDNA from defecated material. It is likely that different ranges of eDNA particle sizes exist per species, and across seasons within species^20^. Therefore, samplers looking to maximize the capture of the most abundant particle sizes across species or environments generally opt for small filter sizes^23^. However, smaller filters also capture additional non-target material, which can dilute or inhibit the intended eDNA target^31^. Knowledge of the size and distribution of the optimal eDNA particle size for sampling and genetic analyses are key variables to consider when designing an eDNA based study. Additionally, studies should consider the genetic region used to identify eDNA particles, as the genetic state of eDNA in the natural environment is composed of nuclear and mitochondrial DNA, which differ in their structure, abundance and availability^32–34^.

Whether eDNA exists primarily as mitochondrial (mtDNA) or nuclear (cDNA) DNA is unclear. Presently, many eDNA studies utilize genetic tools to target mitochondrial genes due, in part, to existing sequence databases being predominately based on the COI mitochondrial gene^3^. Additionally, mtDNA is expected to occur in higher density compared to cDNA since each cell has 2–10 mitochondria per cell, compared to 2 copies of cDNA inhabiting the nucleaus^35^. However, tandemly repeated ribosomal cDNA-based genes, including the small subunit (18S) and the large subunit (28S), occur at similar or higher copies per cell compared to mtDNA^34,36^. From forensic-based research there are known benefits to utilizing short nuclear ribosomal markers, due to higher PCR amplification success of degraded ribosomal cDNA^33,37^. Recent eDNA studies have also suggested that ribosomal gene markers are optimal for differentiating closely related species^38^, and for increased detectability of fish species from eDNA samples, compared to mtDNA markers^34^. However, mtDNA has a more stable cellular structure compared to ribosomal cDNA, rendering mtDNA likely better for detecting and assessing the fate of eDNA over longer time spans^33^.

The fate (i.e. persistence) of eDNA in aqueous environments varies between hours and a month, depending on the system being sampled and the underlying abiotic and transport dynamics associated with the sampled ecosystem^39^. Fate dynamics of eDNA are perhaps the most studied, yet one of the least understood aspects of aqueous eDNA research due to the complex dynamics involved in lentic^40,41^, lotic^42,43^ and marine environments^44^. In general, once released into an environment, eDNA starts to decay or leave the system, approximately following a first order exponential decay function, due to abiotic conditions (temperature, oxygen, pH)^43^, transport factors (flow rate, turbidity)^15,45^, substrate absorption (substrate and biofilm)^46^ and possibly biotic interactions (microbes and extracellular enzymes)^20^. Additionally, variations in physical properties of DNA, such as length, conformation, and structure, alter the susceptibility of eDNA decay to abiotic and biotic factors^20^. Less understood is whether various states of eDNA including particle size or nuclear versus mitochondrial sources degrade similarly under common environmental conditions.

**Figure 1:**
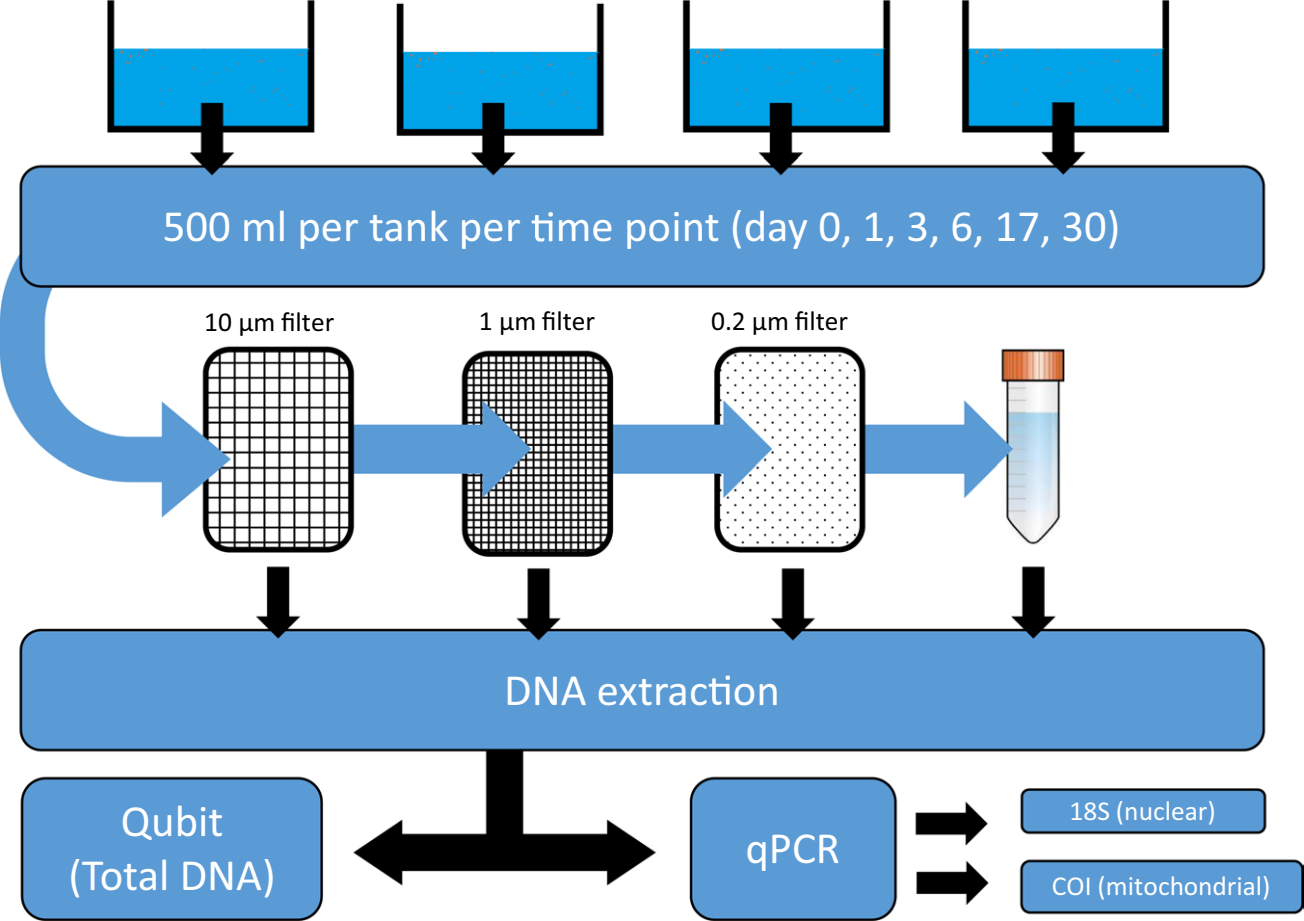
Overview of the study design from sampling to DNA quantification and qPCR amplification and analysis. The four tanks (mesocosm) used in the experiment (3 treatments plus 1 control) are depicted at the top of the figure. The subsequent workflow is depicted following the arrows downwards whereby 500 ml samples were collected from each tank at days 0, 1, 3, 6, 17 and 30, which were sequentially filtered through three filter sizes 10, 1 and 0.2 μm with the final filter effluent water retained and stored as depicted with a conical tube. All four samples sizes (3 filters and effluent water) were extracted separately and each extract was quantified for total DNA using a Qubit fluorometer and quantified using qPCR using *Daphnia magna* specific primers targeting the 18S and COI gene regions.

Only by knowing the state of macrobial eDNA in the environment will we be able to select the most efficient eDNA sampling method. In this study we conducted a mesocosm experiment (Fig. 1) to assess the size distribution (state) and temporal dynamics (fate) of aquatic macrobial eDNA particles originating from mitochondrial and nuclear genetic fragments. Using eDNA originating from *Daphnia magna* we addressed three main objectives: (i) quantify the eDNA particle size distribution of eDNA using filter size sorting; (ii) compare eDNA quantifications between mitochondrial and nuclear derived material via quantitative PCR (qPCR); (iii) calculate the decay rate across detectable eDNA particle size classes for mitochondrial and nuclear derived eDNA. Collectively, this study enhances the understanding of the state of macrobial eDNA in aquatic environments to assists researchers in adapting eDNA sampling and analytical methods.

## Results

**Figure 2:**
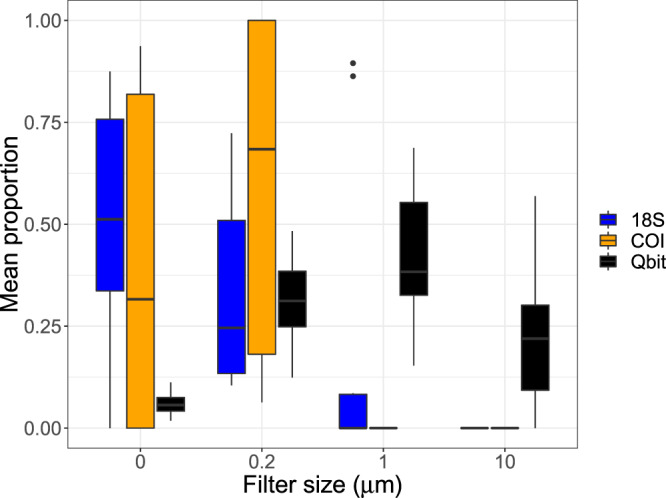
Mean proportional (to compare across 18S, COI and Qbit values) of eDNA quantities (y-axis) from sampling time points (days) 0, 1, 3 and 6 among filter sizes (x-axis). Shown are the values from the 18s (blue) and COI (orange) eDNA qPCR quantification and total DNA (black) from the Qbit quantification. For each boxplot N = 12, corresponding to 3 replicate samples per time point. The upper and lower whiskers correspond to the 1.5 times interquartile range.

**Figure 3:**
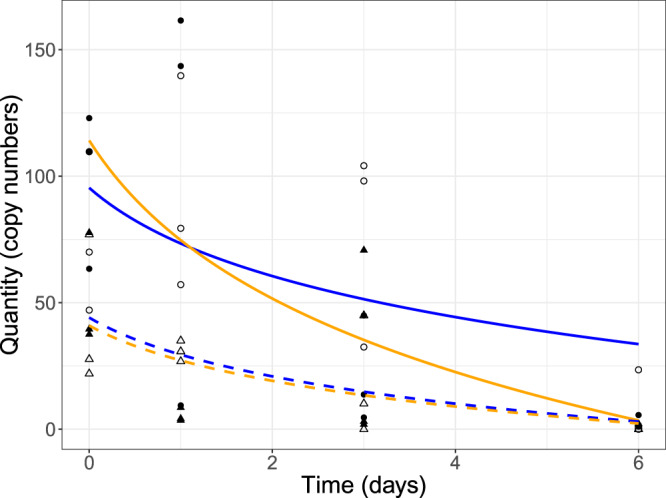
Logarithmic curve fits (lines) and individual sample copy numbers (points) for 18S (solid lines, open points) and COI (dotted lines, black points) for 0.2 μm (orange line, circles) and water effluent (blue line, triangles) from time 0 to day 6. The y-axis shows the copy number as derived from the qPCR analysis against sampling time on the x-axis for time points 0, 1, 3 and 6. Other time points were not included due to limited (day 17) or no (day 31) amplification.

Mean starting (day 0) eDNA concentrations, quantified via qPCR, were 75.61 ± 31.67 copy numbers (copies) for the 18S water effluent (size 0), 98.77 ± 31.30 copies for 18S 0.2 μm filter, 51.58 ± 22.63 copies for COI water effluent, and 42.19 ± 0.30.25 copies for COI 0.2 μm filter. Total DNA concentrations, quantified via Qbit fluorometer, were lowest in the water effluent 0.544 ± 0.504 ng/ul compared to 3.02 ± 3.28 for the 0.2 μm filters, 4.15 ± 6.92 in the 1 μm filers and 2.66 ± 4.32 in the 10 μm filters (Fig. 2). The negative controls samples showed zero amplification across all filter sizes, replicates and time points. Successful amplification (across all replicates) from water effluent and 0.2 µm samples were recorded for both mitochondrial (COI) and nuclear (18s) markers for days 0, 1 and 3. Environmental DNA decay occurred primarily from day 0 to day 6 (one week) for COI and 18S markers for all water effluent and 0.2 µm filter samples, with inconsistent amplification occurring for day 17 and no amplification occurring for day 31 (Fig. 3). Amplification generally failed for samples collected on the 1 µm and 10 µm filters, for both COI and 18S markers, apart from isolated amplification occurring up to day 6 for the 1 µm and 10 µm filters (Fig. 2).

From our repeated measures ANOVA, which included an AR1 correlation structure to account for the temporal autocorrelation for each tank across time^47^, we found significant effects of filter size (p-value < 0.001) and marker (p-value = 0.022) on eDNA copy number quantification, and a non-significant filter x marker interaction (p-value 0.124) (Table 1). The model employed had the lowest AIC score of all alternative models, indicating a better model fit over alternative models, including a linear regression model that excluded the AR1 correlation structure (Table 2). Decay rates differed primarily among filter types within the 18S amplified samples, with a decay rate of −0.360 per day for the effluent water samples versus −0.544 per day for the 18S 0.2 µm filter samples and −0.628 for COI effluent water samples versus −0.501 per day for the COI 0.2 µm filter samples (Table 3, Fig. 3).

**Table 1:** Repeated measures ANOVA statistics for eDNA quantification as the response variable with filter size (Filter) and gene marker region (Marker) as explanatory variables. An AR1 residual correlation structure was used to account for the temporal dependance about the repeated measures for each tank replicate per time point.

	num DF	Sum Sq	Mean Sq	F-value	p-value
Filter	3	26146	8911	10.87	<0.001
Marker	1	6317	4383	5.35	0.022
Filter x Marker	3	5477	1603	1.96	0.124

**Table 2:** Summary of the best models to explain the variation in eDNA quantity related to filter size and gene marker (18S and COI). The best model is indicated in bold based on AIC model comparisons values. We also show comparisons with the null model and against the full linear model, which excluded the AR1 residual correlation structure.

Model	df	AIC
Quantity ~ Filter * Marker	**10**	**1322**
Quantity ~ Filter + Marker	7	1341
Quantity ~ Filter	6	1349
Quantity ~ Marker	4	1383
Null Model	3	1387
Full Linear Model	9	1384

**Table 3:** Exponential decay rates derived from the 18S and COI markers eDNA quantifications from day 0 to day 6.

Marker	Filter	Exp growth (decay) rate
18s	0	−0.360
18s	0.2	−0.544
COI	0	−0.628
COI	0.2	−0.501

## Discussion

Our findings show that eDNA fate followed an exponential decay function. We found that the eDNA particle size for *D*. *magna* is predominantly between 0 and 0.2 µm, suggesting eDNA state is predominately subcellular in nature, meaning cell organelles or free-floating DNA strands. Furthermore, nuclear derived eDNA quantifications were greater than mitochondrial derived eDNA quantifications, suggesting nuclear eDNA was more readily available for capture in our environmental samples.

Degradation of eDNA in our study occurred primarily over a week, with highly variable eDNA detection persisting for up to a month. Under natural environmental conditions eDNA is detectable up to a month in lentic systems^40^, around 24 hours in the lotic system^19,^ and ranging from hours^43^ to months under experimental settings^48^. Additionally, the persistence of eDNA across different species has been experimentally shown to vary from hours to months^28,31,49^. Hence, the rate of eDNA decay observed was comparable to previous studies, particularly for eutrophic environments^44^ where biotic interactions are suspected to promote eDNA degradation rates^20^. We did observe algal growth within the mesocosms overtime, likely facilitated by the constant 20 °C climate, which suggested that biotic activity could have played a role in degradation^20^. Additionally, the pH of the water source, though not measured here, is categorized as moderately soft (low mineral), which infers neutral to slightly acidic pH (~6.5–7). The low pH, which has been found to promote eDNA degradation^43^, may also explain the eDNA decay observed here, which is similar to rates of eDNA decay observed at neutral and slightly acid pH conditions in other lentic experimental studies^50,51^. However, further testing is required to verify the exact mechanisms of eDNA degradation in our particular system. We also observed unequal degradation rates for different size eDNA particles, specifically for the 0.2 µm and 0 µm (water effluent) particle size fractions, whereby decay was greater over time for the 0.2 µm size fraction compared to the 0 µm size. As suggested by Turner *et al*. 2014, the breakdown of sub-cellular structures (0.2 µm) into free nucleotide strands (0 µm) is likely prolonging the decay of the 0 µm size class, thereby allowing the extended observation of the smaller size class through the extension of natural degradation processes^28^.

Total copy numbers from the 18S derived nuclear eDNA samples were ten times greater than the COI derived mtDNA eDNA, indicating nuclear eDNA was more readably amplified from our eDNA samples. The temporal decay dynamics and size fractioning (i.e. state) were similar for the 18S and COI derived eDNA, indicating that nuclear eDNA was generally more abundant, but that eDNA detectability was not affected by nuclear or mitochondrial origin. In general, technical performance differences among PCR-based genetic markers/primers is greatly associated with GC content and melting temperature, however these aspects should be tested in-silico during the primer design stage and do not reflect differences observed in this study. Specifically, the primers utilized in this study have similar melting points. The GC content is lower in the COI marker despite the 18S marker having greater performance, when higher GC content is expected to reduce PCR performance due to the higher GC bond strength^31^. The underlying differences between the COI and 18S markers observed in this study are possibly due to nuclear eDNA being more sensitive to eDNA detection compared to mitochondrial eDNA^34,36^. Forensic based molecular biology suggest that nuclear DNA may be more readily available for PCR amplification, while also degrading more rapidly compared to mitochondrial DNA^33^. The increased degradation rate and increased availability of nuclear DNA, are possibly due to the chromatin structure of nuclear DNA being more susceptible to exonucleases and protease compared to the more protective and less accessible circular structure of mitochondrial DNA^33,52^.

Animal mitochondria range from 0.2 to 1.2 µm in diameter, with length between 1–8 µm^25^. Our findings suggest that given the lack of eDNA detection from the 1 and 10 µm filters, that the primary state of accessible eDNA originating from *D*. *magna* was subcellular. At the start of the experiment, the most abundant eDNA particle size was on the 0.2 µm filters, for both nuclear and mitochondrial derived eDNA. In contrast, the water effluent samples (size 0 µm) generally retained the highest amount of eDNA over time, which can be associated with free-floating nucleotide strands. The high initial quantity of 0.2 µm eDNA combined with the longer persistence of smaller fragments exhibits a distinct temporal dynamic of eDNA particle state and fate. Aqueous macrobial eDNA typically originates from excreted urine, faeces, cellular by-products, and epidermal tissues. Once released from its bodily source eDNA initially exist as large/bulk particles (>1000 µm)^28^. However, large particles readily break apart due to physical (e.g. water currents, abrasion) and environmental factors, such as temperature, oxygen, pH, salinity, light, and decomposition via microbial and extracellular enzymatic activity^24^. Potentially, once released and exposed to the environment, decaying cellular matter rapidly degrades due to hydrolysis and apoptosis destroying cell walls^53^. Subsequently, apoptotic released mitochondria, ribosomes and other subcellular material accumulate, due to endonucleases resistance of intercellular structures, thereby prolong the persistance of short eDNA fragments^54,55^. In aqueous environments the double membrane of the mitochondria protects the mitochondrial cell and inner mitochondrial nucleoid from rapid lysis, whereas nuclear DNA decays more rapidly due to its chromatin structure exposing it to exonuclease activity^56^.

*Daphnia magna* eDNA was primarily captured on the 0.2 µm filter and from the water effluent, whereas a paucity of *D*. *magna* eDNA was capture on the larger 1 µm and 10 µm filters. The only two previous size sorting experiments found eDNA predominantly on filters of larger pore size (1–1.2 µm)^26,28^. The overarching conclusion from previous studies, was that fish sourced eDNA was of a cellular nature and likely comprised of loosely aggregated cells and tissue, which are captured on filters with pore sizes greater than 1 µm^26,28^. Why then the differences between the *D*. *magna* and fish eDNA capture studies? Both of the previous experiments utilized fish sourced eDNA, specifically Common carp (*Cyprinus carpio*) and Brook trout (*Salvelinus fontinalis*) at high eDNA concentrations (i.e. fish biomass to water volume), which may have included sampling of fish tissue or eDNA material released as a result of animal stress. Conversely, our study did not include *D*. *magna* in the experimental tanks and relied on a diluted source of eDNA, thereby avoiding the likelihood of filter clogging from non-targeted fragments. Two working hypotheses can then be postulated to address the subtle, yet important differences between the present and previous studies. 1) The nature of eDNA particles from fish and macroinvertebrate taxa differ in their size distributions. Whole mitochondrial cells range between 0.2 µm and 1.2 µm in diameter with length between 1–8 µm, and would be captured primarily by the 0.2 µm. Whereas, eukaryotic cellular structures (>0.8 µm) are larger than the 0.2 µm filter sizes and would accumulate on 1 or 10 µm filters^25^. Fisheries derived eDNA may feature larger agglomerations of cellular material, thereby explaining the preferential captured efficiently on 1 µm filters^26,28^. Conversely, invertebrate eDNA, originating from smaller-bodied individuals compared to fish, may consist primarily of fragmented epithelial cells or mitochondrial DNA. 2) Alternatively, the nature of the eDNA in the two previous and our present studies are of a similar size distribution, however the volume of eDNA and organic particulates was greater in previous studies that utilized eDNA from larger biomass (fish) and water sourced from natural ponds and streams. Such that the suspended particulate matter and increased eDNA concentration in Turner *et al*. (2014) and Wilcox *et al*. (2015) may have clogged the larger filter, which allowed subcellular eDNA to be captured on the 1 µm filter. Further experiments, exploring different levels of sediment loading, or particulate organic matter could distinguish between the two hypotheses, providing definitive insights into the genuine state of macrobial eDNA.

There is a general need to understand the reliability of eDNA-based detection methods^3,31,57^. Environmental DNA fate and state, as presented here, encompasses two core components required to enhance future eDNA-based studies^58^. Knowledge of the state of eDNA in natural environments, including particle size distribution and the predominantly available gene, are important for efficient capture of eDNA material and for modelling eDNA dynamics^28^. Furthermore, if eDNA sampling design fails to account for the correct eDNA state there is increased potential for false negatives, which can compromise research efforts. Our study suggests that sampling frequency, filter size selection, and decay dynamics play a significant role in the detection of eDNA particles, though such variables are likely to be influenced by the background environment or the study species. Specifically, we show that efficient capture of invertebrate eDNA from water samples requires smaller pore sizes than previously suggested. The larger implications of our findings to wider eDNA research being that eDNA particle state differs among species or environmental conditions, which need to be considered for effective eDNA study design. Overall, our findings yield much needed insights into the state, fate and dynamics of eDNA, with clear implications and guidance for future eDNA research aiming to improve species detection and quantification.

## Methods

### Experimental setup and sampling

*Daphnia magna* were cultivated for two weeks in mesocosms (initial density ~200 individuals/L) at Bangor University, UK. Animals originated from a single clone provided by Birmingham University. Environmental DNA-rich water from *D*. *magna* was collected by sieving individuals from the water using a 45 μm sieve into 3 sterilized 10L tanks containing 10L unchlorinated tap water (the same water used to culture *D*. *magna*), to serve as experimental replicates. We did not collect water chemistry for the water, which is categorized as moderately soft (low mineral), which infers a below neutral pH (~6.5–7). A fourth tank filled with 20L unchlorinated tap water served as the experimental control, which serves to detect contamination during the course of the experiment. Replicate and control tanks were placed in a walk-in climate controlled room set to 20 °C throughout the experiment. The climate room was located in a separate room and building from where the *D*. *magna* was cultivated and sieving of *D*. *magna* did not occur in the climate-controlled room to avoid potential contamination and to ensure no live animals were included in the experiment. Lids were placed on the tanks and only removed when sampling occurred for each tank. Water samples (500 ml) were taken at 0, 1, 3, 6, 17 and 31 days after the start of the experiment. For each 500 ml sample, we sequentially filtered the water through 10, 1, and 0.2 µm filters and retained 50 ml of the final filtered effluent water (size 0) for eDNA extraction. If filters clogged during filtration, additional filters were used such that 500 ml of water was passed through each filter size. Filters and water effluent samples were immediately placed in −20 °C for subsequent DNA extraction (Fig. 1). Filters were stored in 15 ml conical tubes while water effluent samples were stored in 50 ml conical tubes. No additional preservatives were added to the samples prior to storages at −20 °C. Extractions were performed for all samples after the experiment was completed.

### DNA extraction and qPCR analysis

DNA was extracted from both the filter and retained water samples. For DNA extracted from filters, we used a modified Qiagen blood and tissue extraction protocol^59,60^. DNA from water samples were first concentrated by adding 3M sodium acetate (10% per water sample volume) and ethanol (2:1 ethanol to sample volume) then storing at −20 °C for 2 hours. Concentrated samples were then centrifugation at maximum speed for 30 min to form a pellet and the effluent removed^61^. DNA was then extracted from the pellet using the standard Qiagen blood and tissue kit following the manufacturer’s protocol.

Quantification of extracted eDNA was performed using COI and 18S species-specific targeted qPCR assays (Table 4), developed by Primer Design Ltd (Southampton, UK). As the primers were purchased by a company, the exact design methods were not disclosed, however we can provide a brief overview of the general methods employed to generate the primer sets. In short, reference sequences for the targeted gene regions are queried for potential amplicons between 50–150 bp (e.g. using NCBI primer blast). Potential amplicon sequences are then assessed in-silico for species specificity by querying a sequence database with each potential amplicon. Once suitable amplicons are found the respective primers and probes are tested against template DNA originating from the species of interest (here *D*. *magna*) to verify amplification. It is important that any primer set be assessed for specificity within a given study. For the purposed of this experiment, we confirmed that the primers were not successful in amplifying algae originating from biofilm samples in the climate control or cultivation rooms. Furthermore, the control tanks, which utilized the same source water as the treatment tanks, served as direct comparison against non-targeted amplification. Triplicate reactions were performed for each sample and primer combination. Each 20 μL reaction contained 1 μL primer/probe mix (300 nM), 10 μL (2X) PrecisionPLUS Mastermix (Primer Design Ltd.), 2 μL DNA extract, and 7 μL DNAse-free water. Reactions were run on a QuantStudio Flex 6 Real-Time PCR System (Applied Biosystems, USA) with the following protocol: 2 min at 95 °C, followed by 40 cycles of 10 s at 95 °C and 60 s at 60 °C. For the standard curve, 363 and 341 bp synthesized gBlock fragments (Integrated DNA Technologies Inc.) were designed for the COI and 18S primer sets, respectively, that encapsulated the targeted amplified regions of the aforementioned primer pairs. For each qPCR plate, a five-fold dilution of the appropriate standard curve dilution was added, including 1, 10, 100, 1000, 10000 copies. We also quantified the total DNA collected for each sample using a Qubit fluorimeter (Invitrogen, Waltham, USA).

**Table 4:** Sequence details for the primers and probes used for the qPCR amplification of *D*. *magna* eDNA samples.

Target region	Primer/Probe	Sequence (5′ to 3′)	Length	Amplicon length	Tm	GC%	Reference
COI	Sense	TCGGAATGATCTCTCATATTATCAGTC	27	101 bp	56.7	37	YP_009133115.1
	Antisense	ACCTAAGACACCAATAGCTAATATAGC	27				
	Probe	TCCCAAAGGCTTCCTTCTTCCCTCTTTCG	29				
18S	Sense	AGGATGGGSGAGTTGGTGT	19	128 bp	56.9	52.4	AM490278
	Antisense	TGACGACGACCGAGAAACAA	20				
	Probe	CTTGCGTTTCCTGCCGGGCTGC	22				

In addition, we tested qPCR inhibition by spiking control samples from time points 1, 3 and 31(days) for each filter size (water effluent, 0.2 µm, 1 µm, 10 µm) as well as all samples at time day 31, which did not amplify for any samples. We added 2 ul of our 10000 size standard to 18 ul of each tested sample, resulting in a one-step dilution, which we then compared to the CT value of the 1000 size standard. Inhibition in the control samples spiked size standard was negligible (<3% divergence)^31^ with percent divergence being on average 0.633% (SD = 0.56%) among control tanks, 0.973% (SD = 0.415%) among time 31 tanks and 0.962% (SD = 0.435%) across each filter size. We also checked COI inhibition by spiking control tank samples, as above, but were limited due to low remaining extraction volume, but still found negligible inhibition with percent divergence for across control tanks being 1.25% (SD = 0.117) and across filter types being 1.69% (SD = 1.25).

### Statistical analysis

For statistical analyses, we did not include eDNA quantities where no amplification occurred across the group (filter sizes 1 µm and10 µm and days 17 and 31) to avoid zero inflation of the data and potential type I errors. All statistical analyses employed R, version 3.5.1^62^. To assess the effect of filter size and gene marker choice (i.e. the response variables) on DNA quantification (i.e. the explanatory variable), we used a repeated measures ANOVA via the gls function in the nlme package, with a AR1 correlation structure to account for the temporal autocorrelation for each tank across time (i.e. the repeated measure)^47^. Generalized least squares (gls) allows within group residual structuring to directly model variance-covariance structure for the response by extending the ordinary least squares model used in linear regression^47^. The gls residuals structuring is particularly useful for dealing with temporal or spatial autocorrelation, which can lead to type I errors when unaccounted for in linear regression models^63^. The AR1 implements an autoregressive correlation structure for a sequential set of integers in a supplied vector, which makes it suitable for structuring residual spreads over times^64^. We assessed the fitness of the gls model by comparing the fitted model to the null and parsimonious versions of the model as well as alternative fitted linear model using AIC^47^. Additionally, we assessed temporal dynamics of the water effluent and 0.2 µm filter data over time using a first order decay model for each set of filter and gene marker data combination^65^.

## Data Availability

Data associated with the study are available on Figshare; 10.6084/m9.figshare.9699143.
