# Author Correction: Environmental DNA size sorting and degradation experiment indicates the state of *Daphnia magna* mitochondrial and nuclear eDNA is subcellular

**DOI:** 10.1038/s41598-021-95567-6

**Published:** 2021-08-04

**Authors:** Rashnat Moushomi, Gregory Wilgar, Gary Carvalho, Simon Creer, Mathew Seymour

**Affiliations:** Molecular Ecology and Fisheries Genetics Laboratory, School of Natural Sciences, Bangor University, Bangor, Gwynedd LL57 2UW UK

Correction to: *Scientific Reports* 10.1038/s41598-019-48984-7, published online 29 August 2019

This original version of this Article contained errors.

In the legend of Figure 2,

“Shown are the values from the 18s (red) and COI (green) eDNA qPCR quantification and total DNA (blue) from the Qbit quantification.”

now reads:

“Shown are the values from the 18s (blue) and COI (orange) eDNA qPCR quantification and total DNA (black) from the Qbit quantification.”

In the legend of Figure 3,

“Exponential decay fits (lines) and individual sample copy numbers (points) for 18S (solid lines, open points) and COI (dotted lines, black points) for 0.2 μm (orange line, circles) and water effluent (blue line, triangles) from time 0 to day 6.”

now reads:

“Logarithmic curve fits (lines) and individual sample copy numbers (points) for 18S (solid lines, open points) and COI (dotted lines, black points) for 0.2 μm (orange line, circles) and water effluent (blue line, triangles) from time 0 to day 6.”

In Table 4, the third column heading,

“Sequence (3′ to 5′)”.

now reads:

"Sequence (5′ to 3′)”.

The original Article has been corrected.

